# Spirituality-Based Intervention in Hypertension: EFfects on Blood PrEssure and EndotheliaL Function—FEEL Trial Results

**DOI:** 10.5334/gh.1390

**Published:** 2025-01-21

**Authors:** Maria Emília Figueiredo Teixeira, Weimar Kunz Sebba Barroso, Andréa Araújo Brandão, Ana Luiza Lima Sousa, Roberto Esporcatte, Mário Henrique Elesbão de Borba, Ana Clara Neri Ávila Baleeiro, Beatriz Caldas Gonçalves, Enzo Inumaru, Enzo Mata de Sousa, Giovana Barros Leal, Henrique Soares de Araújo Pereira Farias, Juliana Alves de Souza, Lure Emilly Barreto da Silva, Matheus Canguçu de Paiva Queiroz, Frederico Rafael Moreira, Priscila Valverde de Oliveira Vitorino, John Eikelboom, Álvaro Avezum

**Affiliations:** 1 Hypertension Unit, Cardiology Section, Medical School, Federal University of Goiás, Brasil; 2 Federal University of Goiás, Brasil; 3 Spirituality and Cardiovascular Medicine Department, Brazilian Cardiology Society –DEMCA/SBC, Brasil; 4 Medical School, Federal University of Goiás, Brasil; 5 Albert Einstein Hospital, Goiânia, Goiás, Brasil; 6 Department of Chest Diseases, University of the State of Rio de Janeiro, Brasil; 7 School of Nursing, Federal University of Goiás, Brasil; 8 Cardio Clínica do Vale, Lajeado, RS –Brasil; 9 School of Medical and Life Sciences, Pontifical Catholic University of Goiás, Brasil; 10 Medical School, Federal University of Goiás, Brasil; 11 Medical School, University Center of Mineiros, Brasil; 12 International Research Center, Hospital Alemão Oswaldo Cruz, Brasil; 13 School of Social and Health Sciences, Pontifical Catholic University of Goiás, Brasil; 14 Population Health Research Institute, McMaster University, Canada

**Keywords:** Spirituality, Hypertension, Pulse Wave Velocity, Vascular Endothelium, Randomized Controlled Trial

## Abstract

**Background:**

Emerging evidence suggests that spirituality improves patient outcomes, however, this has undergone only limited evaluation in randomized trials. Hypertension is a major cause of cardiovascular morbidity and mortality worldwide.

**Objectives:**

To evaluate whether a spirituality-based intervention, compared to a control group, can reduce blood pressure (BP) and improve endothelial function after 12 weeks in patients with mild or moderate hypertension (HTN).

**Methods:**

Open randomized controlled trial of adults with stage I or II hypertension. Following baseline evaluation, including lifestyle questionnaires, and measurements of office and central blood pressure (BP), home blood pressure monitoring (HBPM) and flow mediated dilation (FMD), patients were randomized to a spirituality-based intervention, which included training for forgiveness, gratitude, optimism, and life purpose delivered by daily WhatsApp communications, or to the control group (CG). Main outcomes were between group difference in change from baseline to 12 weeks in office and central BP, HBPM and FMD, using t-tests, analyses of covariance (ANCOVA) adjusting for baseline differences, and, in addition, missing data imputation as a sensitivity analysis.

**Results:**

Fifty-one patients were randomized to spirituality-based intervention and 49 to control group. Baseline characteristics were well balanced between groups. Spirituality training, compared with control, improved 7.6 mmHg office systolic blood pressure (SBP), 4.1 mmHg central SBP and 4.1 percentage points FMD. Compared to control group, t-test demonstrated statistical significance for office SBP (–7.04 mmHg, p = 0.047) and FMD (7.46 percentage points, p < 0.001), and ANCOVA adjustment for baseline differences showed statistical significance for central SBP (–6.99 mmHg, p = 0.038) and FFMD (7.95 percentage points, p < 0.001) There was no significant effect on HBPM.

**Conclusion:**

A spirituality-based intervention was associated with improved control of office SBP and FMD. These findings will be prospectively evaluated in a nationwide larger and well-powered RCT.

## Introduction

Spirituality has been currently defined as ‘a construct of moral, mental, and emotional values that guide an individual’s thoughts, behaviors, and attitudes in both intra- and interpersonal relationship, amenable to scientific evaluation through proper methodology’ (1). Although often utilized as a therapeutic tool in clinical medicine, high quality evidence supporting the efficacy of spirituality as an intervention is lacking (2).

Arterial hypertension (HTN) is a major cause of morbidity and mortality worldwide, significantly impacting life expectancy, quality of life, and the global economy (3,4). Despite the proven benefits and affordability of blood pressure-lowering medications, HTN often remains poorly controlled (5) with adverse consequences for the vessels, heart, brain, and kidneys (6). Additional non-pharmaceutical interventions that improve blood pressure (BP) and outcomes would be desirable (7).

Spirituality-based interventions that stimulate edifying feelings and reduce the harmful ones have shown promise (8,9,10) but have undergone only limited evaluation in randomized trials. We performed a randomized trial to evaluate the efficacy of a spirituality intervention for treatment of HTN. Our specific objectives were to determine whether, in patients with mild or moderate HTN, a spirituality-based intervention compared with control reduces BP and improves endothelial function after 12 weeks.

## Methods

### Study design and participants

The trial methods have been published previously (11) and the trial is registered at ensaiosclinicos.gov.br (RBR-7 m7ct53). Briefly, FEEL was an open randomized controlled trial, evaluating the use of a spirituality-based intervention in stage I and II hypertension. The trial was approved by the institutional review board from the Clinical Hospital from Federal University of Goias (registration number: 5.487.621), and all patients provided written informed consent. Trial was reported according to Consolidated Standards of Reporting Trials (CONSORT) 2010 updated statement (12). The trial was coordinated by and performed at the Hypertension Unit, Federal University of Goiás, Brazil, a HTN reference center, and was scientifically supported by the Spirituality and Cardiovascular Medicine Department of the Brazilian Cardiology Society (DEMCA/SBC).

### Eligibility criteria

Patients were eligible if they had been previously diagnosed with hypertension, stage I (Systolic blood pressure (SBP) 140–159 mmHg, and/or diastolic blood pressure (DBP) 90–99 mmHg) or II (SBP 160–179 mmHg and/or DBP 100–109 mmHg), with or without antihypertensive medication. Patients were excluded if office BP was ≥ 180/110 mmHg, any changes had been made to antihypertensive therapy in previous 30 days, or the patient was unable to receive or read messages via WhatsApp.

### Randomization

Patients were randomized using a 24-hour, centralized automated, internet-based system (www.randomizer.org) in a 1:1 ratio to receive spirituality-based intervention or control (routine care only).

### Data collection

#### Patients’ assessment

All participants were evaluated in two office visits, being the first (V1) at the beginning of the study and the final (V2) after ± 3 days of the 12-week intervention period. No changes were made to regular medication and lifestyle habits (physical exercise practice, alcohol intake, or smoking) during the inter-visit interval for the patients who completed all the study procedures.

One follow-up phone call was scheduled at 42 days after V1, to ask about symptoms, reinforce the maintenance of lifestyle habits and medication, and, for experimental group, encourage adherence to activities.

#### History and physical examination

At the first visit (V1), all participants underwent a standardized cardiology consultation, including recording of demographics (age, sex), medical history (diabetes, dyslipidemia, other), lifestyle, including smoking (never, current smoking or former smoking), regular alcohol intake (no or yes, but no quantity was specified), physical activity (no, yes irregular, when <150 minutes/week, yes regular, when >150 minutes/week), medications (dosage, frequency) and religion (Catholic, Protestant, Spiritist, other) and performance of anthropometric measurements (weight, height, and body mass index).

#### Measurement of blood pressure and flow mediated dilation

Office BP was measured three times, one minute apart with an OMROM HBP1100, according to Brazilian hypertension guidelines (13). The average of the two last measurements was used to determine office BP.

Central BP was measured with a triple shot protocol using a Cardios Arteris@ device in C1 calibration, obtaining peripheral SBP (SBPp) and DBP (DBPp), central SBP (SBPc) and DBP (DBPc), pulse wave velocity (PWV) and augmentation index (AiX) corrected for heart rate. The averages of the three measurements were calculated for these variables (14).

Flow-mediated dilation (FMD) was assessed by only one investigator, to minimize examiner bias (15), with the gold standard device Unex EF38, which uses Doppler ultrasound attached to an articulated robotic arm to measure the diameter of the brachial artery before and after ischemia caused by inflating a cuff 50 mmHg above the individual’s SBP for five minutes (16). Increases of 10% or more in basal diameter of the artery are considered normal. Assessment of FMD was carried out due to its relevance as an outcome for short-term non-pharmacological interventions (17).

All research subjects were instructed on how to perform the Home Blood Pressure Monitoring (HBPM) using an OMRON HEM-9200T device, according to the Brazilian guideline (14). Over the next four days, BP was measured three times, one minute apart, in the morning and evening, always with an empty bladder, and before meals or taking antihypertensive medication.

### Intervention procedures

Subjects randomized to the spirituality-based intervention received a daily WhatsApp message interspersed with rest days to facilitate adherence (for schedule, see Table 1). Each message required no more than five minutes to complete. Messages included a short video, a written message for self-reflection, or an activity based on content addressed in the previous video. Messages were aimed at stimulating forgiveness, ‘considered a process of releasing resentment’ (18); gratitude, defined as recognizing and responding with kindness ‘to the roles of other people’s benevolence in the positive experiences and outcomes that one obtains’ (19); optimism, ‘defined as a stable, generalized expectancy for the occurrence of good outcomes in life’ (20); and purpose in life, ‘defined as a self-organizing life aim that stimulates goals, manages behavior, and provides a sense of meaning’ (21).

**Table 1:** Schedule of messages’ content for the intervention group.


DAY	TASK	DAY	TASK	DAY	TASK	DAY	TASK

1	Video 1	22	Activity 6	43	Day off	64	RM 22

2	RM 1	23	RM 8	44	Video 9	65	Activity 21

3	Activity 1	24	Day off	45	RM 15	66	Day off

4	Day off	25	Video 6	46	Activity 14	67	Video 12

5	Video 2	26	RM 9	47	RM 16	68	RM 23

6	RM 2	27	Activity 7	48	Activity 15	69	Activity 22

7	Activity 2	28	RM 10	49	RM 17	70	RM 24

8	Day off	29	Activity 8	50	Activity 16	71	Activity 23

9	Video 3	30	Day off	51	Day off	72	Day off

10	RM 3	31	Video 7	52	Video 10	73	Video 13

11	Activity 3	32	RM 11	53	RM 18	74	RM 25

12	Day off	33	Activity 9	54	Activity 17	75	Activity 24

13	Video 4	34	Activity 10	55	RM 19	76	RM 26

14	RM 4	35	RM 12	56	Day off	77	Day off

15	Activity 4	36	Activity 11	57	Video 11	78	Video 14

16	RM 5	37	Activity 12	58	RM 20	79	RM 27

17	Day off	38	Day off	59	Activity 18	80	RM 28

18	Video 5	39	Video 8	60	RM 21	81	Day off

19	RM 6	40	RM 13	61	Activity 19	82	Video 15

20	Activity 5	41	Activity 13	62	Day off	83	RM 29

21	RM 7	42	RM 14	63	Activity 20	84	Activity 25


RM: reflection message.

Adherence was evaluated by WhatsApp read receipts (two blue ticks) for all messages and, for activities, received answers were also considered.

The videos contained a script written by the research group and narrated in a neutral tone by a professional voice actress and neutral graphic art developed by a professional graphic designer.

Messages for self-reflection were quotes related to the topic, all from known authors, properly referenced.

Activities included answering questions related to the content of the videos or completion of related tasks: for example, preparing and sending a message to someone to be forgiven, or writing down personal life purposes.

Participants received all messages from the same investigator number. They were informed that no conversation could take place between them, and no doubts could be cleared, to avoid investigator bias.

## Statistical analysis

### Sample size calculation

With 35 individuals per group, the study was designed to have 90% statistical power to detect a difference of 3 mmHg in the mean BP between Intervention and Control groups, assuming a common standard deviation of 3.82 mmHg, using a two-sided unpaired t-test with significance level (alpha) of 5% and allocation ratio 1:1 (22). The sample size calculation was performed using the EZR (version 1.64) (23) statistical software application for R (24). Considering accommodating for a maximum drop-out rate of 30%, the total sample size was increased to 100 individuals (50 per group) (22).

### Statistical analysis

Baseline characteristics were reported as counts and percentages for categorical variables and means and standard deviations (SD) for normally distributed variables. Differences between randomized groups at baseline characteristics were assessed using unpaired two-sample t-tests for quantitative variables and using chi-square test or Fisher exact test, when appropriate, for categorical variables (25,26,27).

Intergroup comparisons were assessed using unpaired two-sample t-tests for all quantitative outcomes (28,29,30). These outcomes were defined as absolute differences between 12 weeks (final visit) value minus baseline (initial visit) value. Analysis of covariance (ANCOVA) was also performed while controlling for the effects of gender and BMI (baseline) (28,29,30). The adjusted means (with 95% CI) were reported for ANCOVA models. All assumptions for ANCOVA, including homogeneity of regression slopes, equality of variances and normality of residuals, were checked and met (28,29,30,31,32,33). Normality assumptions for ANCOVA was assessed by visual inspection of histogram plot and Q-Q plot of residuals and application of Shapiro-Wilk (SW) normality test (30,31,32,33). The assumption for homogeneity of regression slopes was assessed by rerunning the ANCOVA models, but this time including covariate-independent variable interactions (30,33). The assumption of homogeneity of variance for unpaired two-sample t-test and ANCOVA was evaluated with Levene’s test (30,31).

Intragroup comparisons were assessed by two sample paired t-tests (28,29,30). The assumption that paired differences should be normally distributed was evaluated using plots (Q-Q plot and histogram) and SW test (28,30,31,32).

Additionally, as a sensitivity analysis, statistical analyses were also performed after conducted missing imputation using a machine learning model-based imputer (simple tree) approach. This requires a model to be created for each input variable that has missing values. The default model is a 1-NN (single nearest neighbor) learner (34,35). The Machine learning methods are known to be the best to impute missing data to improve accuracy (36).

All hypothesis tests were two-sided and a p-value < 0.05 was considered statistically significant. Statistical analyses were performed using R 4.2.3 (R Foundation, Vienna, Austria) (37), Jamovi v2.5.6 (38) and JASP v0.18.3.0 (39).

## Results

From 07/27/2022 through 11/25/2022, 100 patients were enrolled (49 in the control and 51 in the intervention group). The baseline characteristics of participants were relatively well balanced between the groups (Table 2). Mean age was 57.3 (SD 11.5) years, 71 (71%) were women and most prevalent risk factors were sedentary lifestyle (47%), alcohol intake (25%) and current smoking (12%).

**Table 2:** Baseline characteristics.


	INTERVENTION (N = 51)	CONTROL (N = 49)	p-VALUE

**Gender**, n (%)			0.011^1^

Male	9 (17.6%)	20 (40.8%)	

Female	42 (82.4%)	29 (59.2%)	

**Diabetes**, n (%)			0.728^1^

No	37 (72.5%)	34 (69.4%)	

Yes	14 (27.5%)	15 (30.6%)	

**Physical activities**, n (%)			0.427^1^

No	23 (45.1%)	24 (49.0%)	

Yes—irregular	16 (31.4%)	10 (20.4%)	

Yes—regular	12 (23.5%)	15 (30.6%)	

**Marital status**, n (%)			0.193^2^

Single	10 (19.6%)	11 (22.4%)	

Married	28 (54.9%)	18 (36.7%)	

Living together	6 (11.8%)	4 (8.2%)	

Divorced	4 (7.8%)	10 (20.4%)	

Widow	3 (5.9%)	6 (12.2%)	

**Smoking**, n (%)			0.794^1^

Never	39 (76.5%)	35 (71.4%)	

Former	6 (11.8%)	8 (16.3%)	

Current	6 (11.8%)	6 (12.2%)	

**Religion**, n (%)			0.885^2^

Protestant	22 (43.1%)	21 (42.9%)	

Catholic	20 (39.2%)	22 (44.9%)	

Spiritist	2 (3.9%)	1 (2.0%)	

Others	7 (13.7%)	5 (10.2%)	

**Alcohol intake**, n (%)			0.299^1^

No	36 (70.6%)	39 (79.6%)	

Yes	15 (29.4%)	10 (20.4%)	

**Age**			0.204^3^

Mean (SD)	55.8 (10.6)	58.8 (12.3)	

**BMI**			0.043^3^

Mean (SD)	32.0 (5.9)	29.7 (5.4)	

**Office SBP**			0.470^3^

Mean (SD)	130.1 (15.3)	127.9 (15.1)	

**Office DBP**			0.418^3^

Mean (SD)	79.6 (10.9)	77.9 (8.8)	

**Central SBP**			0.220^3^

Mean (SD)	117.5 (12.9)	114.4 (12.6)	

**Central DBP**			0.960^3^

Mean (SD)	81.8 (10.6)	81.9 (9.5)	

**HBPM SBP**			0.765^3^

Mean (SD)	122.5 (10.8)	123.3 (12.6)	

**HBPM DBP**			0.765^3^

Mean (SD)	80.6 (9.6)	80.1 (7.3)	

**PWV**			0.302^3^

Mean (SD)	8.0 (1.5)	8.3 (1.7)	

**FMD**			0.773^3^

Mean (SD)	9.8 (5.1)	10.2 (6.1)	


^1^P-value from Chi-Square test;^2^P-value from Fisher Exact test;^3^P-value from unpaired two-samples t-test with equal variance. BMI—body mass index, SBP—systolic blood pressure, DBP—diastolic blood pressure, HBPM—home blood pressure monitoring, PWV—pulse wave velocity, FMD—flow mediated dilation.

**Figure 1:**
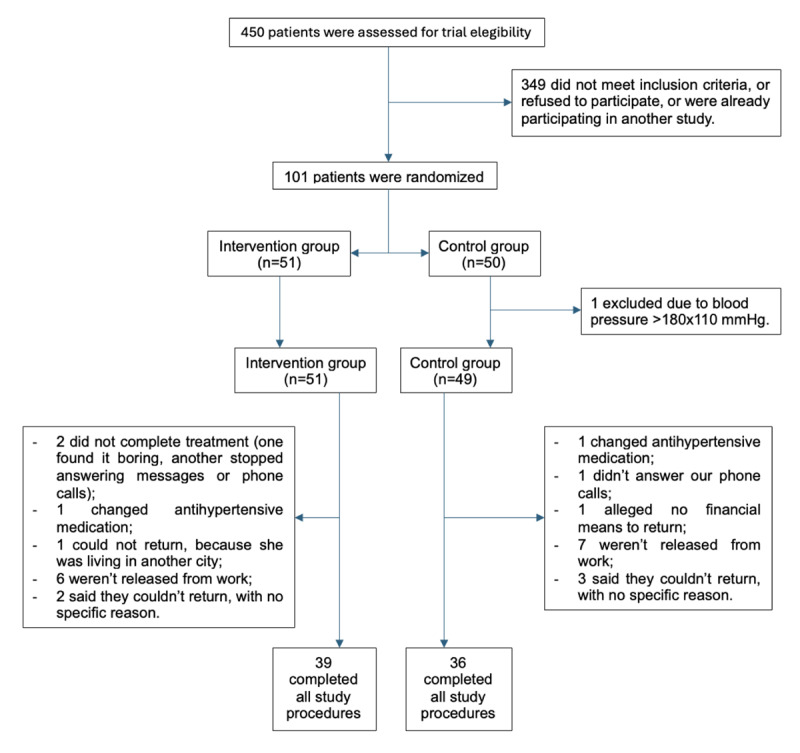
Flow diagram of randomized patients, beginning to end of the study.

Of the 100 patients randomized, 75 participants completed both baseline and end of study BP and FMD measurements and contributed to the primary analyses (Figure 1), accordingly to the previous sample size calculation. As the timeframe for the final visit was strict (±3 days after the 12-week intervention period), this can explain part of the high dropout rate. This and all other reasons are detailed in Figure 1.

The main results are presented in Tables 3 and 4. Intervention group had significant improvement in the office SBP, central SBP and FMD, while control group had a worsening of office DBP and FMD. When these differences were compared between the groups, office SBP and FMD showed statistically significant benefit in the spirituality-based intervention group and, when adjusted for gender and BMI, office DBP, central SBP and FMD were statistically improved in the spirituality-based intervention group by the end of the follow-up period. Missing data imputation strengthened and improved previous results, demonstrating statistically significant benefits for office and central SBP, office DBP and FMD (Tables 5 and 6).

**Table 3:** Study outcomes: within group comparisons between first and final visits.


OUTCOME	SPIRITUALITY-BASED INTERVENTION GROUP (G1) n = 39					CONTROL GROUP (G2) n = 36				
	
	MEAN	SD	LOWER 95%CI	UPPER 95%CI	P-VALUE	MEAN	SD	LOWER 95%CI	UPPER 95%CI	P-VALUE

**Office SBP (V2)**	121.37	13.09				126.63	15.55			

**Office SBP (V1)**	128.97	15.60				127.18	15.30			

**Office SBP (V2-V1)**	–7.60		–11.72	–3.48	< 0.001	–0.56		–6.40	5.29	0.848

**Office DBP (V2)**	80.58	10.83				81.65	8.39			

**Office DBP (V1)**	79.39	10.08				77.28	9.08			

**Office DBP (V2-V1)**	1.19		–1.72	4.10	0.412	4.38		1.84	6.92	0.001

**Central SBP (V2)**	113.42	11.79				115.12	13.97			

**Central SBP (V1)**	117.52	12.84				113.74	12.33			

**Central SBP (V2-V1)**	–4.11		–7.87	–0.34	0.034	1.38		–3.66	6.41	0.582

**Central DBP (V2)**	81.15	10.79				80.38	10.37			

**Central DBP (V1)**	81.82	10.19				81.53	9.47			

**Central DBP (V2-V1)**	–0.67		–3.17	1.82	0.588	–1.14		–4.72	2.43	0.520

**HBPM SBP (V2)**	121.69	10.27				122.31	12.87			

**HBPM SBP (V1)**	123.13	10.34				121.92	12.51			

**HBPM SBP (V2-V1)**	–1.44		–4.02	1.15	0.268	0.39		–1.93	2.70	0.735

**HBPM DBP (V2)**	80.56	10.16				79.81	6.38			

**HBPM DBP (V1)**	80.87	9.25				79.39	7.94			

**HBPM DBP (V2-V1)**	–0.31		–1.86	1.25	0.691	0.42		–1.37	2.20	0.638

**PVW (V2)**	7.77	1.24				8.42	1.61			

**PWV (V1)**	7.82	1.37				8.28	1.69			

**PVW (V2-V1)**	–0.05		–0.22	0.13	0.584	0.14		–0.07	0.35	0.179

**FMD (V2)**	14.28	6.67				7.23	5.71			

**FMD (V1)**	10.16	5.24				10.57	6.31			

**FMD (V2-V1)**	4.12		1.41	6.82	0.004	–3.34		–6.36	–0.33	0.031


SD – standard deviation, V1 – initial visit, V2 – final visit, SBP - systolic blood pressure, DBP - diastolic blood pressure, HBPM – home blood pressure measurement, PWV – pulse wave velocity, FMD – flow mediated dilation.

**Table 4:** Study outcomes: between groups comparison.


OUTCOME	GROUP	n	UNADJUSTED ANALYSIS VIA t TEST				ADJUSTED ANALYSIS VIA ANCOVA		
	
			MEAN	SD	95% CI	p-VALUE^1^	MEAN	95% CI	p-VALUE^2^

**Office SBP (V2 – V1)**	Control	36	–0.56	17.26	(–6.4; 5.29)	0.047	–0.38	(–5.56; 4.81)	0.063

	Intervention	39	–7.60	12.71	(–11.7; –3.48)		–7.37	(–12.96; –1.79)	

**Office DBP (V2 – V1)**	Control	36	4.38	7.51	(1.83; 6.91)	0.102	4.49	(1.72; 7.27)	0.031

	Intervention	39	1.19	8.98	(–1.72; 4.1)		0.14	(–2.85; 3.13)	

**Central SBP (V2 – V1)**	Control	36	1.38	14.88	(–3.65; 6.41)	0.078	1.77	(–2.71; 6.25)	0.038

	Intervention	39	–4.10	11.62	(–7.87; –0.34)		–5.00	(–9.82; –0.17)	

**Central DBP (V2 – V1)**	Control	36	–1.15	10.56	(–4.7; 2.43)	0.824	–1.06	(–4.13; 2.00)	0.684

	Intervention	39	–0.68	7.71	(–3.17; 1.82)		–1.96	(–5.26; 1.34)	

**HBPM SBP (V2 – V1)**	Control	36	0.39	6.84	(–1.93; 2.70)	0.293	0.68	(–1.84; 3.20)	0.579

	Intervention	39	–1.44	7.98	(–4.02; 1.15)		–0.32	(–3.04; 2.39)	

**HBPM DBP (V2 – V1)**	Control	36	0.42	5.27	(–1.37; 2.2)	0.535	0.52	(–1.20; 2.25)	0.722

	Intervention	39	–0.31	4.80	(–1.86; 1.25)		0.09	(–1.77; 1.94)	

**PWV (V2 – V1)**	Control	36	0.15	0.62	(–0.06; 0.36)	0.155	0.17	(–0.03; 0.37)	0.134

	Intervention	39	–0.05	0.55	(–0.22; 0.13)		–0.04	(–0.25; 0.17)	

**FMD (V2 – V1)**	Control	36	–3.34	8.90	(–6.36; –0.33)	<0.001	–3.01	(–5.95; –0.07)	<0.001

	Intervention	39	4.12	8.35	(1.41; 6.82)		4.94	(1.77; 8.11)	


^1^p-value from unpaired two-samples t-test with equal variance; ^2^p-value from ANCOVA models adjusted for gender and BMI (baseline). SD – standard deviation, V1 – initial visit, V2 – final visit, SBP - systolic blood pressure, DBP - diastolic blood pressure, HBPM – home blood pressure measurement, PWV – pulse wave velocity, FMD – flow mediated dilation.

**Table 5:** Study outcomes: within group comparisons between first and final visits (missing imputation analyses).


OUTCOME	SPIRITUALITY-BASED INTERVENTION GROUP (G1) n = 51					CONTROL GROUP (G2) n = 49				
	
	MEAN	SD	LOWER 95%CI	UPPER 95%CI	P-VALUE	MEAN	SD	LOWER 95%CI	UPPER 95%CI	P-VALUE

**Office SBP (V2)**	121.95	11.67				125.05	13.80			

**Office SBP (V1)**	130.14	15.29				127.93	15.15			

**Office SBP (V2-V1)**	–8.19		–11.85	–4.52	< 0.001	–2.88		–7.83	2.07	0.248

**Office DBP (V2)**	81.09	10.02				82.67	8.40			

**Office DBP (V1)**	79.57	10.92				77.95	8.84			

**Office DBP (V2-V1)**	1.52		–1.01	4.06	0.234	4.72		2.74	6.70	<0.001

**Central SBP (V2)**	114.11	10.56				114.80	12.15			

**Central SBP (V1)**	117.54	12.91				114.40	12.59			

**Central SBP (V2-V1)**	–3.43		–6.88	0.01	0.051	0.40		–3.71	4.52	0.845

**Central DBP (V2)**	80.86	9.49				80.46	9.02			

**Central DBP (V1)**	81.80	10.60				81.91	9.50			

**Central DBP (V2-V1)**	–0.95		–3.32	1.42	0.426	–1.44		–4.35	1.46	0.324

**HBPM SBP (V2)**	121.79	10.63				123.24	12.44			

**HBPM SBP (V1)**	122.60	10.73				123.28	12.46			

**HBPM SBP (V2-V1)**	–0.8		–2.94	1.34	0.455	–0.04		–1.96	1.88	0.965

**HBPM DBP (V2)**	80.51	9.44				79.90	5.77			

**HBPM DBP (V1)**	80.54	9.50				80.15	7.28			

**HBPM DBP (V2-V1)**	–0.03		–1.42	1.37	0.968	–0.25		–1.70	1.21	0.735

**PVW (V2)**	7.95	1.33				8.45	1.60			

**PWV (V1)**	7.97	1.47				8.30	1.72			

**PVW (V2-V1)**	–0.02		–0.17	0.13	0.769	0.15		–0.03	0.32	0.103

**FMD (V2)**	14.08	6.22				7.10	5.01			

**FMD (V1)**	9.83	5.13				10.15	6.12			

**FMD (V2-V1)**	4.25		1.95	6.56	<0.001	–3.05		–5.46	–0.65	0.014


SD – standard deviation, V1 – initial visit, V2 – final visit, SBP - systolic blood pressure, DBP - diastolic blood pressure, HBPM – home blood pressure measurement, PWV – pulse wave velocity, FMD – flow mediated dilation.

**Table 6:** Study outcomes: between groups comparison (missing imputation analyses).


OUTCOME	GROUP	N	UNADJUSTED ANALYSIS VIA t TEST				ADJUSTED ANALYSIS VIA ANCOVA		
	
			MEAN	SD	95% CI	p-VALUE^1^	MEAN	95% CI	p-VALUE^2^

**Office SBP (V2 – V1)**	Control	49	–2.88	17.24	(–7.83; 2.07)	0.085	–2.457	(–6.858; 1.944)	0.045

	Intervention	51	–8.19	13.03	(–11.8; –4.52)		–8.959	(–13.775; –4.143)	

**Office DBP (V2 – V1)**	Control	49	4.72	6.91	(2.735; 6.70)	0.050	4.869	(2.549; 7.19)	0.019

	Intervention	51	1.52	9.03	(–1.01; 4.06)		0.828	(–1.712; 3.367)	

**Central SBP (V2 – V1)**	Control	49	0.40	14.33	(–3.71; 4.52)	0.153	1.09	(–2.645; 4.826)	0.042

	Intervention	51	–3.43	12.25	(–6.88; 0.01)		–4.519	(–8.607; –0.43)	

**Central DBP (V2 – V1)**	Control	49	–1.44	10.11	(–4.35; 1.46)	0.791	–1.114	(–3.758; 1.53)	0.736

	Intervention	51	–0.95	8.43	(–3.32; 1.42)		–1.766	(–4.66; 1.128)	

**HBPM SBP (V2 – V1)**	Control	49	–0.04	6.69	(–1.96; 1.88)	0.598	0.273	(–1.794; 2.339)	0.847

	Intervention	51	–0.80	7.60	(–2.94; 1.34)		–0.019	(–2.28; 2.243)	

**HBPM DBP (V2 – V1)**	Control	49	–0.25	5.07	(–1.7; 1.21)	0.828	–0.035	(–1.483; 1.413)	0.634

	Intervention	51	–0.03	4.96	(–1.42; 1.37)		0.469	(–1.116; 2.054)	

**PWV (V2 – V1)**	Control	49	0.15	0.61	(–0.03; 0.32)	0.146	0.162	(–0.002; 0.327)	0.08

	Intervention	51	–0.02	0.53	(–0.17; 0.13)		–0.05	(–0.229; 0.13)	

**FMD (V2 – V1)**	Control	49	–3.05	8.37	(–5.46; –0.65)	<0.001	–2.738	(–5.137; –0.338)	<0.001

	Intervention	51	4.25	8.18	(1.95; 6.55)		4.587	(1.961; 7.214)	


^1^p-value from unpaired two-samples t-test with equal variance; ^2^p-value from Ancova models adjusted for gender and BMI (baseline). SD – standard deviation, V1 – initial visit, V2 – final visit, SBP - systolic blood pressure, DBP - diastolic blood pressure, HBPM – home blood pressure measurement, PWV – pulse wave velocity, FMD – flow mediated dilation.

One study participant reported anger as the only harm resulted from the intervention.

## Discussion

This is a pilot study, at the forefront of research on this topic. Larger, multicentric studies are already planned. We performed an innovative randomized controlled trial to evaluate the effects of a readily applicable, low cost, spirituality-based intervention on BP and endothelial function in patients with mild or moderate hypertension. The main findings were that the spirituality-based intervention improved SBP control and FMD.

Most previous studies of the possible benefits of spirituality on clinical outcomes are observational. Small, randomized trials have evaluated the effects of meditation (40), psychotherapy (41), and religious interventions (42). Most of them showed a protective effect on human health.

The exact physiological pathway through which the benefits related to the practice of edifying feelings occur is unknown and is likely to be multifaceted. There are hypotheses related to improvements in lifestyle habits, such as reducing smoking, alcohol consumption, drug addiction, or better adherence to healthy diets, exercise practices, or medication treatment (43). None of these hypotheses apply to the group studied here, as all these factors were evaluated by questionnaire at the beginning and end, ensuring that they were not modified during the inter-consultation interval. Another empirical and generical hypothesis encompasses immunological, inflammatory, endocrine, and autonomic mechanisms in the possible cardiovascular modulation (44).

The spirituality intervention that we evaluated focused on four edifying feelings: forgiveness, gratitude, optimism, and life purpose (45,46,47,48). Individuals with a greater capacity for forgiveness, whether innate or acquired through training or therapy have shown benefits in lower left ventricular workload and myocardial oxygen consumption (49), and ultimately lower cardiovascular stress and cardioprotection (50). Gratitude has been related to reduced mental stress, lower cardiovascular risk (51). Optimism has been associated with a lower risk of cardiovascular disease (CVD), lower cardiovascular and all-cause mortality (52). Life purpose or a meaningful life has been linked with lower risk of cardiovascular events and all-cause mortality (53), improve coping and reduce stress in people with CVD (54).

In relation to HTN, multiple mechanisms have been proposed to explain the possible beneficial effects of edifying feelings, mostly related to lower stress levels. Gratitude seemed to work on lowering SBP through a stress-buffering effect on both reactions to and recovery from acute psychological stress (55), and optimism seemed to achieve lower BP by reducing stress intensity and frequency, together with better sleep quality (56). Furthermore, forgiveness, achieved through specific training, seemed to have taken similar paths in HTN treatment, especially in those with elevated levels of anger, probably due to deactivation of sympathetic system, that was previously activated by anger (57). Purpose in life was able to improve BP control, probably because of better sleep, mood, and increase in physical activity practice (58).

The baseline characteristics indicate a population of hypertensive patients with other associated risk factors and mostly adequate BP control. This profile presents a challenge in demonstrating the effectiveness of a potential new treatment strategy to lower blood pressure, especially in the short term. To help detect an effect of our spirituality-based intervention on BP we performed multiple measurements of BP and also measured FMD, which can detect subtle changes in vascular reactivity following short term interventions, mediated by endothelial nitric oxide production (59,60).

It has been previously reported that each percentage point increase in FMD corresponds to a reduction of about 10% in cardiovascular risk (17). The increase of 4.1 percentage points in FMD in the spirituality-based intervention group was greater than that reported with physical activity (61) or increased intake of phytosterols (62).

Limitations to be considered in the current study include a modest sample size, and that 25% did not undergo both baseline and 12-week measurement of BP and FMD, although this was balanced between the groups. This considerable loss happened due to several reasons, such as medications change during follow-up, not being able to return within the preestablished timeframe (±3 days after the 12-weeks intervention period), and for experimental group, not completing all the proposed tasks. Also, this was a single-center study restricted to patients with mild or moderate hypertension. Furthermore, due to the intervention design, total blinding was not possible. Nonetheless, BP was measured with electronic device, and the obtained number was copied directly to the data form, and we adopted a strategy for FMD-examiner blinding. Missing data imputation reinforced the results previously obtained—the intervention group obtained improvements on office and central SBP, plus FMD.

## Conclusion

Spirituality-based interventions that promote edifying feelings can improve both office systolic blood pressure and endothelial function. These results suggest that spirituality-based interventions may be a useful adjunct to standard approaches for adult patients with mild or moderate hypertension, and that these topics could be addressed by physicians with their patients, with potential benefits for cardiovascular health.

## Data Accessibility Statement

The data that support the findings of this study are available, upon request, at https://osf.io/adq5b/?view_only=06326e85b49e40a4a08494ae004a9206, reference number osf.io/adq5b.
